# Supplementary material for: Persistence of Schistosomiasis-Related Morbidity in Northeast Brazil: An Integrated Spatio-Temporal Analysis
Source: Trop Med Infect Dis. 2021 Oct 28;6(4):193. doi: 10.3390/tropicalmed6040193 (PMC8628971; doi:10.3390/tropicalmed6040193)
Supplement: Supplementary file 1 [file tropicalmed-06-00193-s001.zip › tropicalmed-1372064-supplementary.pdf]

Tropical Medicine and Infectious Disease

MDPI

Supplementary Information

Table S1. Number of tests performed and percentage of positivity for schistosomiasis according to state and year in the Northeast Region, Brazil. 2001-2017.

State	Examined (% of positivity)																
	**2001**	**2002**	**2003**	**2004**	**2005**	**2006**	**2007**	**2008**	**2009**	**2010**	**2011**	**2012**	**2013**	**2014**	**2015**	**2016**	**2017**
Maranhão	53,227 (7.2)	72,622 (2.8)	128,186 (4.2)	138,045 (4.2)	171,976 (5.2)	187,701 (4.1)	134,078 (4.0)	90,640 (4.3)	111,879 (4.6)	102,260 (5.0)	93,640 (3.6)	93,236 (2.7)	81,033 (3.5)	82,691 (3.8)	69,340 (3.2)	45,747 (2.5)	43,606 (4.3)
Piauí	8,257 (0.0)	472 (0.6)	627 (0.0)	2,236 (0.0)	132 (0.0)	6,276 (0.1)	6,841 (0.1)	5,426 (0.0)	0 (0.0)	2,189 (0.0)	0 (0.0)	0 (0.0)	0 (0.0)	0 (0.0)	0 (0.0)	0 (0.0)	0 (0.0)
Ceará	103,526 (1.0)	94,627 (1.0)	48,150 (0.8)	42,150 (0.8)	43,573 (0.8)	62,907 (0.4)	62,040 (0.3)	47,684 (0.4)	53,925 (0.3)	41,497 (0.3)	29,316 (0.1)	32,798 (0.1)	26,091 (0.2)	26,498 (0.4)	15,727 (0.8)	0 (0.0)	0 (0.0)
Rio Grande do Norte	5,156 (0.7)	3,357 (1.2)	29,170 (4.8)	22,783 (2.8)	69,376 (7.9)	45,694 (3.7)	61,663 (3.8)	43,807 (3.2)	39,866 (4.5)	104,628 (2.7)	37,267 (2.9)	22,295 (3.8)	34,684 (1.8)	25,671 (2.8)	10,611 (2.3)	3,727 (3.3)	11,612 (2.7)
Paraíba	117,430 (5.0)	107,466 (5.4)	139,516 (5.4)	120,999 (4.9)	117,025 (5.1)	100,725 (4.7)	93,014 (5.4)	53,135 (6.8)	73,146 (6.7)	41,398 (5.0)	47,156 (5.1)	37,306 (5.6)	25,694 (7.9)	23,616 (10.2)	0 (0.0)	0 (0.0)	0 (0.0)
Pernambuco	7,644 (25.4)	18,564 (12.8)	32,059 (16.6)	29,434 (13.2)	70,676 (13.2)	101,286 (11.5)	124,070 (10.3)	88,987 (9.9)	96,756 (9.0)	98,665 (8.3)	103,961 (7.3)	94,544 (5.6)	120,182 (5.4)	257,417 (3.4)	244,844 (3.0)	190,453 (2.3)	176,176 (2.5)
Alagoas	107,752 (16.5)	188,467 (12.9)	224,325 (13.2)	204,768 (10.2)	218,587 (9.6)	244,360 (8.7)	216,903 (8.1)	193,808 (8.4)	185,538 (8.8)	180,994 (7.3)	177,655 (7.3)	166,798 (6.8)	174,090 (7.3)	162,633 (6.0)	158,806 (5.0)	131,097 (5.0)	148,242 (4.7)
Sergipe	53,052 (11.8)	70,518 (10.9)	95,055 (9.5)	92,252 (10.7)	112,771 (9.6)	141,457 (9.9)	131,235 (10.4)	79,858 (10.3)	92,957 (8.8)	91,719 (9.6)	100,387 (8.5)	18,070 (9.8)	50,614 (6.7)	47,058 (6.9)	61,485 (7.4)	29,159 (9.6)	39,776 (7.1)
Bahia	354,111 (6.1)	876,269 (7.1)	717,730 (6.1)	624,394 (6.1)	505,664 (5.1)	465,929 (5.0)	405,405 (4.2)	293,567 (3.5)	292,703 (3.7)	251,217 (4.2)	214,837 (3.3)	128,832 (3.8)	61,592 (2.8)	2,328 (2.6)	0 (0.0)	0 (0.0)	0 (0.0)
Northeast region	810,140 (7.2)	1,432,362 (7.4)	1,414,818 (7.2)	1,277,061 (6.7)	1,309,780 (6.7)	1,356,335 (6.3)	1,235,249 (6.0)	896,912 (5.9)	946,770 (5.9)	914,567 (5.6)	804,219 (5.4)	593,879 (4.9)	573,980 (5.2)	627,912 (4.5)	560,813 (4.0)	400,183 (3.7)	419,412 (3.9)
